# RETRACTED: Dong et al. Inhibition of SDF-1α/CXCR4 Signalling in Subchondral Bone Attenuates Post-Traumatic Osteoarthritis. *Int. J. Mol. Sci.* 2016, *17*, 943

**DOI:** 10.3390/ijms25147757

**Published:** 2024-07-16

**Authors:** Yonghui Dong, Hui Liu, Xuejun Zhang, Fei Xu, Liang Qin, Peng Cheng, Hui Huang, Fengjing Guo, Qing Yang, Anmin Chen

**Affiliations:** Department of Orthopedics, Tongji Hospital, Tongji Medical College, Huazhong University of Science and Technology, 1095 Jiefang Avenue, Wuhan 430030, China; dongyonghuitjmu@gmail.com (Y.D.); liuhui09270808@gmail.com (H.L.); xuejunzhang526@gmail.com (X.Z.); skyfrost444@gmail.com (F.X.); qinliangdoc@gmail.com (L.Q.); pengcheng201507@163.com (P.C.); drulon@icloud.com (H.H.); fjguo@tjh.tjmu.edu.cn (F.G.)

The journal retracts the article “Inhibition of SDF-1α/CXCR4 Signalling in Subchondral Bone Attenuates Post-Traumatic Osteoarthritis” [1] cited above. 

Following publication, concerns were brought to the attention of the publisher regarding a partial overlap of images presented in this article [1].

Adhering to our complaint’s procedure, an investigation was conducted that confirmed a partial duplication with Figure 5a, presenting different experimental conditions. While the authors cooperated with the Editorial Office during the investigation, they were unable to satisfactorily explain the partial overlap or account for anomalies present in the raw material provided. 

As a result, the Editorial Board has lost confidence in the reliability of the findings and has decided to retract this publication [1], as per MDPI’s retraction policy (https://www.mdpi.com/ethics#_bookmark30), accessed on 26 June 2024. 

This retraction was approved by the Editor-in-Chief of the journal *Int. J. Mol. Sci.*

The authors did not provide a comment on this decision. 
